# Autism Spectrum Disorder and Temporal Lobe Epilepsy in a Physician: A Self-Reported Case of Neuropsychiatric and Functional Adaptations

**DOI:** 10.7759/cureus.93350

**Published:** 2025-09-27

**Authors:** Ixequi Luna-Mendoza, Enrique Torres-Rasgado, José Aurelio Cerón-Morales

**Affiliations:** 1 Faculty of Medicine, Benemérita Universidad Autónoma de Puebla, Puebla, MEX; 2 Department of Biostatistics, Benemérita Universidad Autónoma de Puebla, Puebla, MEX; 3 Department of Neurology, Hospital Puebla, Puebla, MEX

**Keywords:** autism spectrum disorder (asd), clinical anxiety, clinical case report, clinical depression, electroencephalography (eeg), neurodiversity, neuroimaging biomarkers, nuclear magnetic resonance, social adaptation, temporal lobe epilepsy

## Abstract

While the comorbidity of autism spectrum disorder (ASD) and epilepsy is well-documented, first-person accounts from physician-patients remain scarce. This report bridges that gap, offering both clinical and functional insights into the lived experience of this dual diagnosis. We detail the case of a 28-year-old physician, a recent medical school graduate, with confirmed ASD level 1 and structural temporal lobe epilepsy (TLE), alongside generalized anxiety disorder and major depressive disorder. The clinical picture is defined by a pronounced sensory hyper-reactivity and a lowered seizure threshold, leading to discognitive and generalized seizures often triggered by sensory or social overload. His management has required a coordinated, multidisciplinary regimen of antiepileptic and psychotropic medications. Our discussion integrates clinical data with a phenomenological perspective. The synergy between ASD and TLE appears to forge a distinct neurobiological profile that demands holistic treatment. The author’s development of functional adaptation strategies, a process of translating neurotypical demands into a neurodivergent operational framework, proved essential for navigating the rigors of medical education. This case illustrates that high professional achievement is attainable for individuals with complex neurodevelopmental comorbidities. It also makes a case for a paradigm shift in medicine and academia toward genuine neuro-inclusion, one that moves beyond simple accommodation to foster environments that truly value neurological diversity.

## Introduction

The confluence of autism spectrum disorder (ASD) and epilepsy is one of the most significant comorbidities in neurodevelopmental medicine. As defined by the Diagnostic and Statistical Manual of Mental Disorders, Fifth Edition, Text Revision (DSM-5-TR), ASD is a neurodevelopmental condition characterized by persistent deficits in social communication and interaction, alongside restricted, repetitive patterns of behavior, interests, or activities. Similarly, epilepsy is a chronic neurological disease defined by an enduring predisposition to generate epileptic seizures and by the neurobiological, cognitive, and social consequences of this condition. Large meta-analyses and systematic reviews confirm that individuals with ASD have a markedly higher risk of developing epilepsy, with prevalence rates estimated between 20% and 30%, a figure that dwarfs the 1% seen in the general population [1,2]. This powerful statistical link points beyond mere coincidence to a shared neurobiological foundation, with current evidence suggesting overlapping mechanisms, including genetic variants that affect synaptic function and a fundamental imbalance between excitatory and inhibitory signaling [3-5]. Clinically, this overlap often culminates in a complex picture where seizure control is deeply intertwined with psychiatric well-being and functional adaptation [6,7].

However, despite this extensive research, the literature remains largely silent on the subjective experience of navigating this dual diagnosis, particularly within the crucible of medical training. Such first-person narratives are invaluable, as they illuminate how abstract neurobiological mechanisms translate into tangible, daily challenges and adaptations.

The perspective of a physician who is also a patient is uniquely instructive. It builds a bridge between objective clinical knowledge and the felt reality of a condition, allowing for a far more nuanced understanding. This report, therefore, offers a detailed clinical description of this comorbidity, examines the interplay between its neuropsychiatric features and the adaptive strategies required to complete a medical education, and ultimately argues for a more holistic, patient-centered model of care for neurodevelopmental disorders.

## Case presentation

This report chronicles the case of a 28-year-old physician who has successfully completed his medical degree and is now awaiting his professional title and license. It details the diagnostic journey, clinical features, therapeutic interventions, and adaptive strategies related to his dual diagnosis of ASD and structural TLE.

The patient has had a neurodevelopmental diagnosis since early childhood, having been first formally diagnosed with Asperger's syndrome at the age of three under DSM-IV criteria. The diagnostic journey detailed in this report began on October 25, 2023, representing a formal reassessment that updated his diagnosis to ASD level 1 in accordance with the modern DSM-5-TR framework. This was confirmed through a semi-structured interview and reinforced by a battery of standardized instruments (including the Modified Checklist for Autism in Toddlers, Revised; Childhood Autism Spectrum Test; Autism-Spectrum Quotient; Social Responsiveness Scale; Autism Diagnostic Observation Schedule; and Adult Asperger Assessment), which all yielded significantly elevated scores. His psychiatric history is complex, with symptoms of anxiety and depression present since age five, manifesting across a wide spectrum from chronic hypervigilance to recurrent panic attacks and severe depressive episodes with anhedonia. Prior to his current care, he was managed by over ten non-specialized psychiatrists, leading to multiple unsuccessful medication trials (including fluoxetine, alprazolam, venlafaxine, and paroxetine); a trial of aripiprazole was notably discontinued due to an acute extrapyramidal reaction.

The diagnosis of TLE was formally established at age 26. However, a retrospective history revealed that subtle seizures, presenting primarily as auditory and tactile hallucinations with depersonalization, often triggered by stress, had been occurring since childhood but had not been previously identified as epileptic events. An exacerbation during his year of social service saw an increase in frequency to 3-4 episodes per week. Routine laboratory investigations were not performed, as there was no clinical suspicion of an underlying metabolic etiology for the seizures.

The functional impact of these symptoms on daily life is significant. Sensory hypersensitivities require constant self-management to avoid overload, such as using polarized lenses for photophobia and noise-canceling headphones for hyperacusis. Alexithymia complicates the self-monitoring of emotional states, increasing the risk of autistic crises. Despite these systemic barriers, the patient developed unique functional adaptation strategies that enabled them to excel academically. He leveraged a capacity for sustained hyperfocus and an excellent photographic memory, allowing him to graduate with a 9.6 GPA, among the highest in his class, and achieve the highest score during his postgraduate rotating internship. He employed strategic social isolation during his university years to preserve cognitive and sensory resources for his studies.

**Figure 1:**
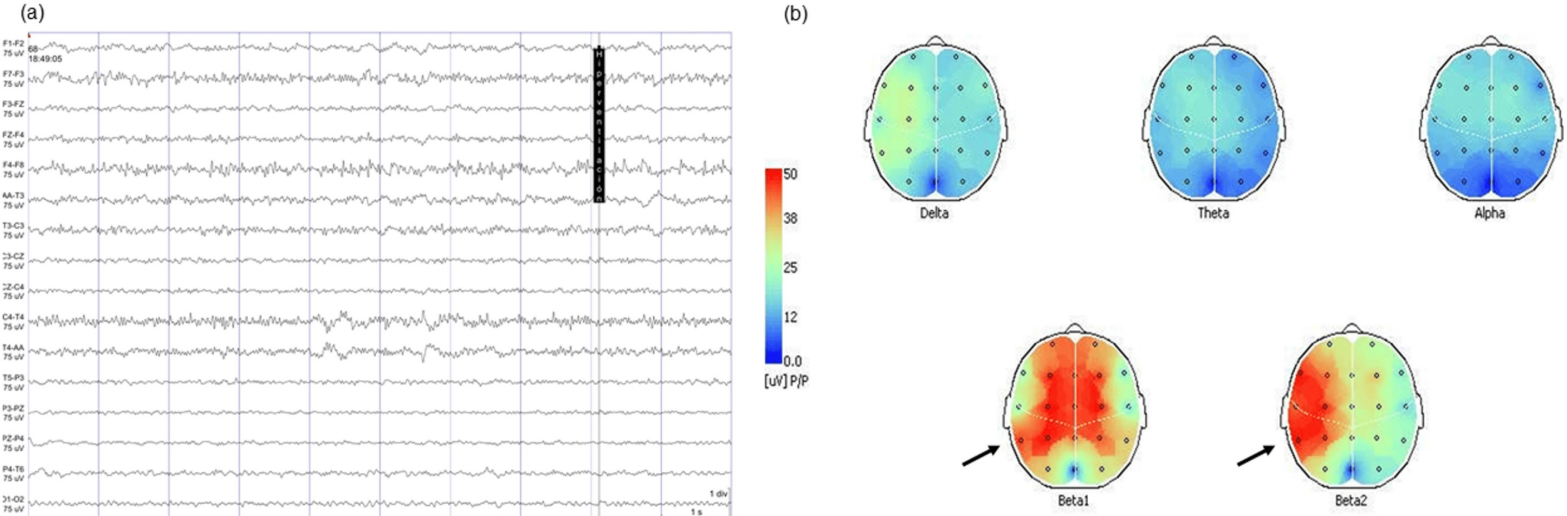
EEG and brain mapping findings This figure illustrates the patient's pattern of cortical hyperexcitability. Panel (a) shows a representative EEG tracing during hyperventilation, notable for intermittent sharp wave activity with a bilateral fronto-temporal predominance. Panel (b) provides a quantitative visualization of this irritative pattern, with topographic maps demonstrating a significant and widespread power increase in the Beta1-2 frequency bands across the fronto-central regions. EEG: electroencephalogram

**Figure 2:**
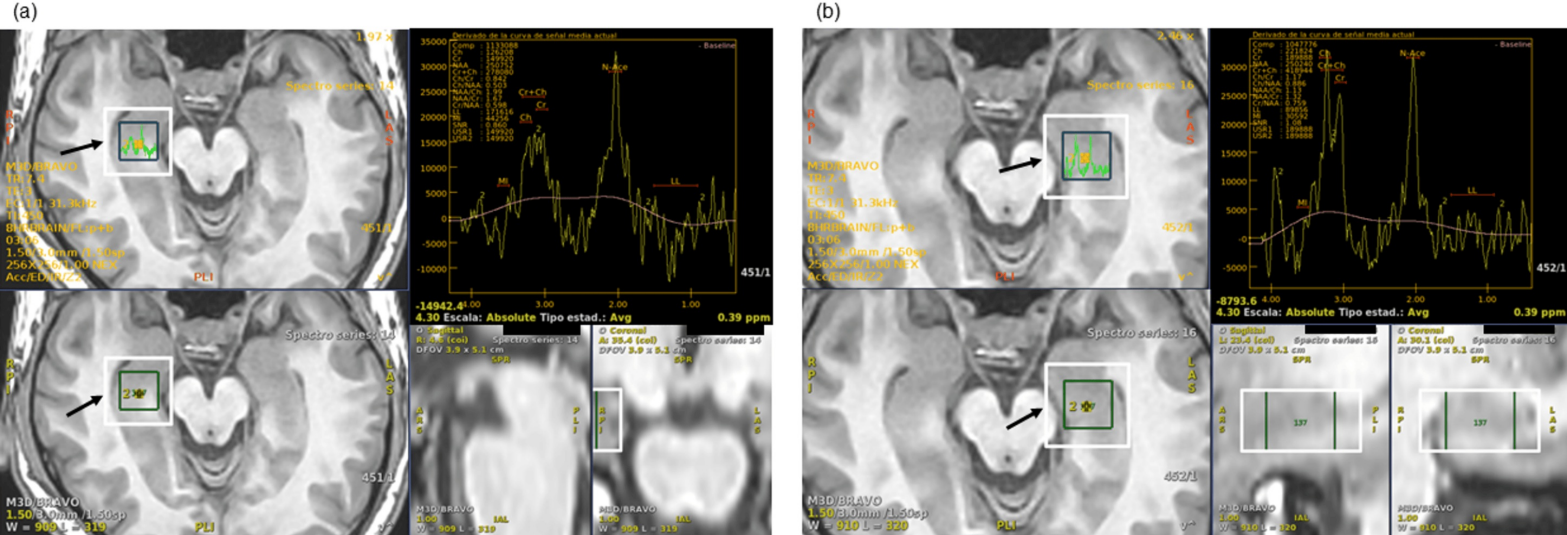
MRS of the temporal lobes Arrows indicate the placement of single-voxel spectroscopy in the right (a) and left (b) temporal lobes. The resulting spectral analysis in both regions revealed a decreased NAA/Cr ratio, a finding consistent with significant bilateral neuronal dysfunction. MRS: magnetic resonance spectroscopy, NAA/Cr: N-acetylaspartate to creatine

The comprehensive workup included an electroencephalogram (EEG), which demonstrated a bilateral fronto-temporal and central irritative pattern, with sharp wave activity facilitated by hyperventilation (Figure 1A) and a predominant power spectrum distribution in the Beta1-2 range (Figure 1B). While conventional MRI sequences appeared largely unremarkable, magnetic resonance spectroscopy (MRS) revealed significant metabolic abnormalities consistent with neuronal dysfunction in both the right (Figure 2A) and left (Figure 2B) temporal lobes.

The patient’s management is based on a permanent, integrated regimen that includes sertraline (150 mg daily), quetiapine (100 mg at night), magnesium valproate (titrated from 1000 mg to 1500 mg daily), and topiramate (50 mg daily). A six-month course of completed transcranial magnetic stimulation supplemented this. He continues with long-term cognitive behavioral therapy, which he has received since age seven, and recently began working with a psychologist specializing in ASD, leading to significant interpersonal improvements. At the time of this writing, his condition is stable. While anxiety and depression scores remain in the high range, they have decreased from the maximum to the minimum threshold for this classification. Functionally, he reports an improved ability to empathize and a deeper intellectual understanding of social cues, enabling him to navigate social situations more effectively.

Taken in its entirety, this case represents a complex clinical phenotype defined by the synergistic interplay of a lifelong neurodevelopmental condition, a structural epilepsy, and significant psychiatric comorbidities. This history is further characterized by a remarkable paradox: the presence of profound functional challenges coexisting with the development of highly effective, atypical adaptive strategies that facilitated exceptional academic achievement. The current stability, achieved through a highly personalized and multimodal therapeutic strategy, provides a compelling foundation for the subsequent discussion on its underlying neurobiology and the broader implications for neurodivergent individuals in demanding professional fields.

## Discussion

This case report is an exercise in neurological cartography from two perspectives: that of the physician who analyzes the map and the patient who inhabits the territory. In my experience, the coexistence of ASD and TLE is not a simple summation of comorbidities but a synergy that redefines perception, cognition, and existence itself. The scientific literature provides coordinates that delineate shared genetic pathways and a fundamental imbalance in cortical signaling [5,6], but only lived experience can reveal the true nature of this inner landscape [8,9].

The pathophysiology described in the literature manifests in my experience as a neurobiological “echo chamber,” where the cortical hyperexcitability of epilepsy appears to amplify the sensory hyper-reactivity inherent to ASD [10]. This insidious feedback loop, in which sensory overload lowers the epileptic threshold, illustrates a clinical phenotype where diagnostic boundaries blur. It demands a therapeutic approach that addresses not two separate conditions, but a singularly integrated neurological system, a concept supported by literature calling for a holistic view of the autism-epilepsy connection [11].

Navigating the diagnostic process as a medical professional revealed a fundamental paradox. On one hand, there was the intellectual trust in objective tools like MRI and EEG. Specifically, the metabolic abnormalities found on MRS provide a functional correlate for the underlying neuronal dysfunction characteristic of a structural epileptic focus, even in the absence of gross anatomical changes on conventional MRI sequences, a finding consistent with advanced neuroimaging literature [12]. On the other hand, there was the visceral confrontation with a healthcare system that, as described by Witchalls et al. [13], often struggles to recognize the nuanced presentations of autism in adults. Diagnosing ASD in adulthood requires a sophisticated approach, relying on retrospective accounts of childhood traits and an understanding of highly developed masking or compensatory strategies. To be both the observer and the observed, the one who understands the DSM-5-TR criteria and the one who embodies them, creates a clinical dissonance that can only be bridged through informed self-advocacy [14].

Pharmacological treatment in this context becomes a delicate act of neurochemical balancing. The use of antiepileptic drugs like magnesium valproate and topiramate extends beyond seizure control; through their mood-stabilizing properties, these agents directly address the psychiatric comorbidities of anxiety and depression, a crucial consideration given their high prevalence in this population [6,8]. This polypharmacy is not a sign of pathology's complexity but rather a testament to a therapeutic strategy that comprehends the profound interconnection between epilepsy, autism, and emotional well-being [15].

Beyond pharmacology lies the domain of functional adaptation, a process this author has come to define as a continuous and metabolically costly act of translation. It involves decoding the implicit expectations of a neurotypical world and re-encoding them into an operational algorithm compatible with a neurodivergent mind. This challenge is central to the cognitive profile of this comorbidity [13]. The specific strategies detailed in the case presentation, such as leveraging hyperfocus as a primary study tool and employing strategic social isolation to preserve sensory resources, were not acts of “overcoming” a deficit. They employed a methodical application of a personalized system of adaptations. This journey mirrors the complex transition to adulthood for many individuals with neurodevelopmental conditions, where integrated care is crucial for long-term success [16].

While this self-report cannot be generalized, its value lies not in statistical power but in its narrative precision. It offers a high-fidelity data point that illuminates meta-analytic findings with the light of lived experience. Future research should therefore prioritize several key areas. First, longitudinal studies are needed to track the long-term efficacy of various therapeutic regimens in this dual-diagnosis population. Second, further investigation into the specific cognitive strengths associated with ASD, such as hyperfocus and pattern recognition, could lead to the identification of novel pedagogical strategies for medical education. Finally, qualitative research that gives voice to the lived experience of neurodivergent physicians is essential. Only by integrating this subjective data with objective clinical findings can we hope to foster a medical culture that is truly holistic, empathetic, and effective.

## Conclusions

The trajectory documented in this report offers a clear conclusion: a complex neurodevelopmental diagnosis, while formidable, does not preclude the highest levels of professional achievement. This case demonstrates that success in such a demanding environment is achieved not by masking or suppressing neurodivergent traits, but rather through a conscious process of functional adaptation. This process involves a deep, personal understanding of one's own neurobiology, which is then used to methodically translate the expectations of a neurotypical world into a sustainable, personalized operational framework.

Consequently, this journey makes a compelling case for a necessary evolution in both clinical practice and medical education. It calls for a definitive shift away from a model of merely accommodating symptoms toward a genuine integration of neurobiological diversity. Such a model would actively dismantle systemic barriers and foster environments that allow the unique cognitive strengths often associated with neurodivergence to flourish. Ultimately, this report serves as a testament to a core principle: that to practice medicine at its most insightful and humane is to recognize diverse ways of functioning not as obstacles to overcome, but as integral facets of the human condition.
